# Primary Genital Herpes Simplex Virus Type I in Preterm Prelabour Rupture of Membranes at 30 Weeks' Gestation

**DOI:** 10.1155/2015/198547

**Published:** 2015-11-15

**Authors:** Anna Dalton, Rosalie Grivell

**Affiliations:** ^1^Women's and Babies Services, Women's and Children's Hospital, North Adelaide, Adelaide, SA 5006, Australia; ^2^Department of Maternal Fetal Medicine, Women's and Children's Hospital, North Adelaide, Adelaide, SA 5006, Australia

## Abstract

*Background.* Disseminated herpes simplex virus (HSV) in the neonate is associated with significant morbidity and mortality. Current guidelines recommend caesarean in third-trimester maternal primary genital HSV outbreaks to prevent transmission from mother to fetus. In the premature fetus, however, expectant management is often necessary to reduce morbidity of prematurity. The benefit of performing caesarean after 6 hrs of rupture of membranes (ROM) to reduce maternal-fetal transmission is unclear.* Case*. A female patient with primary genital HSV type 1 outbreak coinciding with preterm, prelabour rupture of membranes (PPROM) at 30 + 3 weeks' gestation. An immediate caesarean section was not performed after multidisciplinary team discussion due to the benefits of glucocorticoids on immune complications of prematurity. The patient had expectant management for 5 days with intravenous (IV) aciclovir and then delivered an infant vaginally with disseminated neonatal HSV.* Conclusion*. We address the rare presentation of primary HSV infection associated with PPROM and the dilemma of how to manage these patients given the limited literature. We discuss the role of intrauterine compartment monitoring with amniocentesis, the mode of delivery when ROM has occurred for 120 hours, expectant management to reduce prematurity, and the effectiveness of aciclovir to reduce viral shedding in the prevention of neonatal HSV.

## 1. Introduction

Primary HSV infection at the time of delivery is an indication for caesarean to prevent vertical transmission of the virus from mother to fetus. However, in early viable gestation PPROM occurring for more than 6 hours, the protective effects of caesarean are unknown. Furthermore, it is unknown if aciclovir delivered intravenously to the mother confers any immunity for the fetus. In this setting, the significant risk of fetal morbidity and mortality from untreated in utero HSV infection must be balanced against morbidity of prematurity. The best way to deliver these infants after prolonged ROM is also unclear; do we plan for caesarean or vaginal delivery?

## 2. Case Presentation

A 17-year-old gravida-two-para-one woman presented leaking clear fluid per vagina for the last hour and contracting irregularly at 30 + 3 weeks' gestation. She complained of feeling unwell over the last week, with flu-like symptoms and with difficulty urinating for the last two days causing reduced fluid intake. She also described painful areas on her vulva which were new and had been present for 5 days. Her local doctor prescribed antibiotics for suspected urinary tract infection but had not examined her vulva or urine. On arrival to hospital the patient was tachycardic with heart rate 120 bpm but afebrile at 35.6°C. Abdominal examination revealed a longitudinal cephalic fetus, symphysiofundal height 26 cm, mild suprapubic tenderness, and palpable, mild contractions. Sterile speculum examination revealed multiple white, shallow ulcerated lesions on the labia minora, vaginal introitus, and cervix. Rupture of membranes was confirmed with clear amniotic fluid present in the posterior vaginal fornix and positive nitrazine yellow sticks. The cervix was long and posterior with a multiparous external os. Swabs of the vulval lesions were taken for viral polymerase chain reaction (PCR). Cardiotocography (CTG) was reassuring and obstetric ultrasound confirmed oligohydramnios with an amniotic fluid index (AFI) of 2.6 cm. Fetal Doppler scan was normal and the estimated fetal weight was 1537 g on the 24th percentile for weight with symmetrical growth. Her past medical history included smoking 15 cigarettes per day and a previous vaginal delivery at 34 + 5 weeks' gestation in 2012, delivering a female infant weighing 2100 g. She reported no previous sexually transmitted infections; chlamydia/gonorrhoea NAAT, syphilis, Hepatitis C, Hepatitis B, and HIV serology were negative. She had negative HSV types 1 and 2 serology in February 2013. She had not changed sexual partners since the birth of her first child and her partner denied having oral or genital herpes.

## 3. Treatment

The patient was admitted with suspected primary HSV infection (pending lesion swab and blood serology results) and commenced on IV 500 mg aciclovir three times daily (maternal weight based dose), IV benzyl penicillin, oral erythromycin, and oral nifedipine, was steroid covered with two doses of 11.4 mg intramuscular betamethasone given 24 hours apart, and was rehydrated with IV fluid and provided with analgesia. In-out catheter was inserted and she was not in acute urinary retention. Blood tests revealed a white cell count (WCC) of 14 × 10^9^/L and elevated C-reactive protein (CRP) of 26 mg/L. Our institution does not routinely use amniocentesis to detect fetal infection in the setting of confirmed PPROM. Swabs of the vulvar lesions taken at initial examination returned positive for HSV type 1 on viral PCR, 24 hours after admission to hospital; urine culture was negative. After 72 hrs her benzyl penicillin and nifedipine were ceased; she remained on oral erythromycin and IV aciclovir until she delivered. Serology results available 4 days into her admission revealed positive HSV type 1 Immunoglobulin-M (IgM) and negative HSV type 1 Immunoglobulin-G (IgG). HSV type 2 IgM and IgG were also negative; the diagnosis of primary HSV type 1 infection was confirmed serologically. After 72 hours of IV antibiotics and antiviral medication, her WCC remained steady at 15 × 10^9^/L, CRP had dropped to 2 mg/L, and she remained afebrile. A multidisciplinary team of consultant obstetricians, neonatologists, and infectious disease physicians met 4 days after initial presentation and decided to augment delivery as the risk to the fetus in utero with potentially untreated or inadequately treated HSV was greater than the risk of prematurity. At the time of this meeting, serological and PCR results confirming primary HSV infection were available and known. Caesarean was not performed as ROM had occurred for 120 hours and the protective effects of caesarean after more than 6 hrs of ROM are not recorded in the literature. Given that caesarean was unlikely to prevent fetal infection, she was delivered vaginally even in the presence of active herpes lesions. Repeat low vaginal swabs revealed that she was group B streptococcus positive, so at time of augmentation she was recommenced on IV benzyl penicillin as well as continuing IV aciclovir.

## 4. Outcome and Follow-Up

Oxytocin augmentation was commenced at 30 + 6 weeks' gestation and an uncomplicated vaginal birth was achieved 10 hours later. A male infant weighing 1830 g was delivered with a 420 g placenta; Apgar score was 1 at 5 minutes and 9 at 10 minutes. The infant was transferred to the neonatal intensive care unit for resuscitation with continuous positive airway pressure and commenced on IV antibiotics and IV aciclovir for disseminated neonatal HSV infection. Swabs of the neonate were taken 30 minutes after birth and were positive for HVS, suggesting that infection occurred prior to delivery. HSV was detected on all cutaneous swabs, plasma, and urine specimens; however, cerebral spinal fluid was negative. Placental swabs for HSV returned negative. The child was neurologically intact and remained in hospital on IV aciclovir for 21 days and then was commenced on 50 mg oral aciclovir three times daily for 6 months after delivery. Follow-up outpatient appointment was planned for 6 weeks after discharge. The child was followed up through hospital outpatient clinic and diagnosed with developmental delay at age 8 months with predominantly gross motor and expressive speech delay. He continues to be seen by speech pathology, physiotherapy, and the paediatricians.

## 5. Discussion

The prevalence of neonatal HSV ranges from 1 in 3200 to 3500 live births in the United States [1–3]. In Australia, HSV seroprevalence in pregnant women is 14% [4]. The prevalence of primary HSV infection in pregnancy is low, between 0.6 and 2% in United States populations [2, 5, 6]. Although primary HSV infection in pregnancy is uncommon, its diagnosis and adequate treatment are important, as neonatal morbidity from primary HSV in third trimester is as high as 30–50% [1, 4, 5, 7]. This is significantly higher than recurrent HSV infection at the time of delivery, which has a risk of neonatal HSV at 1–3% [5, 7]. The incubation period of HSV is between 2 and 20 days with lesions occurring for up to 21 days [4]. In primary HSV there are no maternal IgG antibodies against HSV to transfer through the placenta to the fetus in utero, and the volume of viral shedding is increased, increasing the risk of vertical transfer [4, 5, 7]. Around 85–90% of neonatal transmission occurs during delivery of the baby, other causes being congenital HSV at 5% and postpartum acquisition in 5–15% of cases [2, 4]. Risk factors for transmission include primary infection, HSV isolated from the cervix, infection with HSV type 1 virus, invasive monitoring, preterm delivery, and maternal age less than 21 years [8]. Neonatal HSV is divided into three categories of disease: skin, eye, and mouth infection; central nervous system disease; and disseminated disease which has the highest rate of mortality (90%) if left untreated [1]. Disseminated disease occurs most commonly with 70% of infants affected and is more likely to occur in preterm infants; approximately 17–20% of these neonates will suffer long-term morbidity including microcephaly, seizures, and mental impairment [5, 6, 9].

Knowing if the fetus is already exposed to HSV within the intrauterine compartment, prior to delivery, could be used to plan management. Amniocentesis can be used to assess for subclinical infection, which occurs in approximately 30% of PPROM cases [10]. However it is best performed as soon as ROM occurs, rather than as a tool to monitor the intrauterine environment during expectant management [10]. Our institution does not routinely use amniocentesis in this setting as we employ empirical antibiotic treatment and most PPROM patients will deliver within 48 hours, and almost all will deliver within one week [10]. In our patient, HSV lesions were present on the cervix, indicating likely ascending infection. Immediate delivery via cesarean would have increased the risks of prematurity such as neonatal death, respiratory distress syndrome, and cerebroventricular haemorrhage [11]. The literature on this topic is limited. Ray et al. describes a case where amniocentesis on intact membranes returned positive for HSV and this resulted in a caesarean with the fetus unaffected [12], suggesting that fetal infection is more complex than just the presence of HSV in the intrauterine compartment. In the setting of unavoidable vaginal delivery in primary HSV, the risk of transmission and subsequent neonatal HSV is 41% [6].

Caesarean delivery in the setting of PPROM is not protective [1]. Caesarean can reduce the exposure to maternal vaginal secretions that contain the virus; however it does not completely eliminate risk, with 86% risk reduction, mainly in a population with primary HSV in which no antiviral treatment had been initiated [2, 7, 8]. Currently there is no consensus on what gestational age the risks of HSV infection outweigh the risks of prematurity [13]. Two case studies report on primary HSV in the setting of PPROM: one at 25 weeks managed expectantly for one week and then delivered by LSCS after labouring; the other PPROM at 24 weeks went onto a term delivery. Both cases were treated with aciclovir and did not result in neonatal HSV [7]. Expectant management is likely to be beneficial between 28 and 32 weeks' gestation [7]; however very premature neonates are at higher risk of perinatal infections [5]. Induction was chosen after one week rather than ongoing expectant management because maternal aciclovir does not guarantee that the fetus will be protected from ascending vertical infection [7, 14, 15]. It was also unlikely that expectant management could have been employed indefinitely in this case, given that protective IgG antibodies take 6–12 weeks to develop [2, 15] and the likelihood of the pregnancy continuing for this long was very low. Although ACOG acknowledges the value of expectant management of PPROM with active HSV, there is no guideline as to how long this should be employed [16].

Finally, the effectiveness of intrapartum aciclovir in reducing neonatal HSV is unknown [6]. It crosses the placenta and becomes concentrated in the amniotic fluid and is excreted by the fetal kidneys [2]. Aciclovir may be less effective in cases of PPROM where amniotic fluid is reduced. The literature suggests that if antiviral therapy has started within 6 days of lesion onset, then shedding duration is reduced from 9 days to 2 days [17]. This suggests our patient was no longer shedding and vaginal delivery would be acceptable.

## 6. Conclusion

Neonatal HSV has significant morbidity and mortality. Understanding the risk factors for transmission is important when trying to avoid infection. However, unavoidable vaginal delivery in the setting of primary HSV results in neonatal infection less than half of the time. In trying to best manage the rare combination of primary HSV and PPROM we must risk stratify prematurity which warrants expectant management, with several other unknown quantities: whether maternal aciclovir given intrapartum to prevent neonatal HSV is effective; whether intrauterine compartment monitoring with amniocentesis can effect neonatal outcome; and whether caesarean after 120 hrs of ROM offers a protective effect. Further evidence on this topic is likely to occur only in the setting of limited case reports and case series, making management of these patients an ongoing dilemma.
